# The Role of Lp-PLA2 as a Mediator Between Serum Magnesium and Zinc Levels and Cardiovascular Risk in Patients With Metabolic Syndrome

**DOI:** 10.7759/cureus.72107

**Published:** 2024-10-22

**Authors:** Khaleel A Manik, P P Sheela Joice, Imran A Jagadal, Jithesh T K, Vijaya Samundeeswari, Basheer Madompoyil, Mohammed Jaffer Pinjar

**Affiliations:** 1 Physiology, Vinayaka Mission’s University, Salem, IND; 2 Physiology, Jawaharlal Nehru Medical College, Belagavi, IND; 3 Biochemistry, Muslim Educational Society (MES) Medical College, Perinthalamanna, IND; 4 Biochemistry, Vinayaka Mission’s University, Salem, IND; 5 Physiology, Al-Azhar Medical College and Specialty Hospital, Thodupuzha, IND; 6 Physiology, All India Institute of Medical Sciences, Deoghar, IND

**Keywords:** atherogenic index, cardiovascular risk, lipoprotein-associated phospholipase a2, metabolic syndrome, micronutrient deficiency, oxidative stress

## Abstract

Background: Metabolic syndrome (MetS) is a collection of conditions that includes abdominal obesity, low high-density lipoprotein (HDL) levels, high triglycerides, hypertension, and impaired glucose metabolism, all of which are risk factors for cardiovascular diseases. Of the biomarkers above, lipoprotein-associated phospholipase A2 (Lp-PLA2) has been highlighted as a critical link between inflammation and the pathogenesis of atherosclerosis, which strongly predicts cardiovascular events. Micronutrients like magnesium and zinc are essential in maintaining metabolic and cardiovascular health, but these micronutrient deficiencies occur frequently among individuals with MetS. This study aimed to consider the association between serum magnesium and zinc levels with Lp-PLA2 and how these associations could link pathways in cardiovascular risk among MetS patients.

Methods: This was a comparative cross-sectional study of 100 cases diagnosed as MetS and compared with an equal number (n = 100) of age and matched healthy control. Blood magnesium, zinc, and Lp-PLA2 levels were determined by colorimetric assay. We also tested the association of Lp-PLA2 with levels of micronutrients, and we evaluated whether Lp-PLA2 was a mediator in the pathway between MetS and cardiovascular risk. The data were analyzed on IBM Corp. Released 2021. IBM SPSS Statistics for Windows, Version 28.0. Armonk, NY: IBM Corp; the results will be considered statistically significant if p < 0.05.

Results: The serum magnesium and zinc concentrations in patients with MetS were significantly lower than in the controls (p < 0.001). The Lp-PLA2 level was much higher in the MetS group than the no-MetS, and it correlated inversely with serum Mg (r = -0.35, p < 0.001) or Zn levels (r = -0.42, p < 0.001). After multivariate analysis, the mediating effect of Lp-PLA2 in the pathway from micronutrient deficiency to cardiovascular risk was maintained, whereby high levels were associated with increased atherogenic index and oxidative stress markers.

Conclusions: These results show that Lp-PLA2 is an intermediate step in the relationship between low levels of some micronutrients and cardiovascular risk among MetS patients. Our findings indicate that a sufficient magnesium and zinc status might offer cardiovascular protection through lessening Lp-PLA2 activity. These observations demonstrate the potential benefits of high-risk enrichment and dietary intervention for detecting and controlling micronutrient deficiencies in MetS subjects to impede further cardiovascular diseases.

## Introduction

The global prevalence of metabolic syndrome (MetS) poses a serious public health challenge, as it is closely linked to cardiovascular diseases (CVD). MetS, an array of conditions consisting primarily of central obesity, dyslipidemia, hypertension, and insulin resistance, is highly associated with CVD, which already remains to be the leading cause of death worldwide [1]. Developing countries have witnessed a steep rise in the prevalence of MetS on account of changes in lifestyle and urbanization, making it perhaps one, if not the significant, healthcare burden for these nations. Understanding the contributors to CV risk in patients with MetS is essential when considering strategies for prevention and management [2].

Magnesium and zinc are trace minerals in various biochemical activities important for cardiovascular health [3]. Magnesium is essential for more than three hundred enzymes regulating glucose metabolism, blood pressure, and vascular tone. CVD is a significant component of cardiometabolic risk since it has been demonstrated that magnesium deficiency correlates with insulin resistance and hypertension and also promotes increased vascular calcification, which is one of the recognized CVD-predicting factors [4]. Zinc, in turn, plays a vital role in maintaining antioxidant defenses, immune function, and cellular repair mechanisms [5]. These micronutrients are also crucial for cardiovascular health (e.g., zinc is notably associated with oxidative stress, and thiamine is involved in energy production) [6].

Lipoprotein-associated phospholipase A2 is an enzyme that primarily localizes on low-density lipoprotein (LDL) particles [7]. These pro-inflammatory molecules can promote plaque development and weaken arterial walls, leading to established plaque rupturing [8]. Increased Lp-PLA2 activity is consistently associated with an increased risk of cardiovascular events (i.e., MI, stroke) and serves as a useful biomarker in the assessment of CVD. However, its association with cardiovascular risk is not entirely independent of LDL levels. Therefore, Lp-PLA2 should be considered in conjunction with LDL levels rather than as an independent predictor of risk [9]. In patients with MetS, Lp-PLA2 is particularly relevant due to its established involvement in the inflammatory processes and atherosclerotic plaque formation that often accompany this condition.

Although the relationship between magnesium, zinc, and Lp-PLA2 with cardiovascular health is established individually, there has been limited understanding concerning how these components interact, especially among MetS [10]. In particular, whether magnesium and zinc deficiencies contribute to Lp-PLA2 activity has not been investigated in detail. This study explores the relationship between Lp-PLA2 and serum magnesium and zinc levels in patients with MetS, hypothesizing that deficiencies in these micronutrients may be linked to an increased risk of elevated Lp-PLA2 levels, potentially influencing cardiovascular events. The research aims to provide a deeper understanding of how micronutrient supplementation could impact cardiovascular risk in MetS patients. The objectives include investigating the association between serum magnesium and zinc levels and Lp-PLA2 activity in MetS patients, comparing these levels between MetS patients and healthy controls, and examining the correlation between Lp-PLA2 levels and other cardiovascular risk factors in MetS patients.

## Materials and methods

A convenient sampling method was employed to evaluate a total of 200 subjects in the study, consisting of 100 patients with Metabolic Syndrome (MetS) and 100 healthy controls. MetS was diagnosed based on the International Diabetes Federation (IDF) criteria, which included central obesity defined as waist circumference >94 cm for men and >80 cm for women, together with two or more of the following factors: increased triglycerides ≥150 mg/dL, low HDL cholesterol <40 mg/dl in males or <50 mg/dl in females, elevated blood pressure: systolic BP≥130 mmHg, diastolic BP≥85 mmHg, and fasting plasma glucose: ≥100 mg/L [11]. No participant met one or more of the following exclusion criteria: severe renal insufficiency, liver disease, malignancies known to induce an acute phase response and alter lipid metabolism, ongoing inflammatory diseases (acute or chronic), treatment with medication that may compromise micronutrient levels, such as antiepileptic drugs or oral contraceptives.

Sample collection

Fasting blood samples were taken from all subjects in the study. Under the aseptic technique, a 5 mL venous blood sample was collected from each subject. Whole blood was allowed to clot and then centrifuged at 3000 rpm for 10 min to harvest serum. The serum was subsequently aliquoted and frozen at -20°C until analyzed.

Measurement of parameters

Standardized biochemical assays were used to measure serum levels of Lp-PLA2, magnesium, and zinc. The serum concentration of Lp-PLA2 was measured using a commercial ELISA kit that explicitly detects and quantifies the enzyme. Atomic absorption spectrophotometry is the most accurate measurement of magnesium and zinc nutrition levels in biological samples. Additional measurements of other cardiovascular markers, including total cholesterol, triglycerides, HDL, and LDL cholesterol, were made to obtain an overall profile precipitated by a cardiorespiratory risk performed with an automated analyzer.

Statistical analysis

The collected data was analyzed using IBM Corp. Released 2021. IBM SPSS Statistics for Windows, Version 28.0. Armonk, NY: IBM Corp. Summary statistics were calculated for all continuous variables (mean value and standard deviation of the mean) as descriptive. The Shapiro-Wilk test was applied to evaluate the data normality. An independent sample t-test was used to compare mean Lp-PLA2, magnesium, zinc, and further cardiovascular markers between MetS patients and healthy subjects. The correlation between serum magnesium, zinc, and Lp-PLA2 was evaluated using Pearson's disorder correlation coefficients. We subsequently performed multivariate regression analysis to assess whether Lp-PLA2 acted as a mediator in the associations of a validated panel of nutrient biomarkers with most cardiovascular risk factors after adjustment for potential confounders.

## Results

There were 200 participants in our study, 100 of whom were MetS patients, and 100 were matched with age and sex-healthy controls. The MetS group participants had a mean age of 46.2 ±8.7 years, while those in the control group averaged 45.8 ±9.1 years (p=0.721). The MetS group had 54 males and 46 females, but the control group consisted equally of 55 men and 45 women (p=0.843). The MetS group had a significantly higher body mass index (BMI) than did the controls (30.1 ±3.4 kg/m² vs. 24.5 ±2.9 kg/m², p <0.001), as per Table 1. This reflected the central obesity present among MetS patients.

**Table 1:** Demographics BMI: Body mass index

Group	Mean Age (years)	p-value (age)	Males (%)	Females (%)	BMI (kg/m²)	p-value (BMI)
Metabolic Syndrome	46.2 ± 8.7	0.721	54	46	30.1 ± 3.4	<0.001
Control	45.8 ± 9.1	0.721	55	45	24.5 ± 2.9	<0.001

Correlation analysis

For patients with MetS, there is a significant inverse correlation between Lp-PLA2 levels and serum magnesium levels (r = -0.35, p < 0.001) as well as zinc ion content in serum (r = -0.42, p < 0.001) as per Table 2. The analysis of the MetS group revealed that the measured levels of these micronutrients are inversely related to the presence of Lp-PLA2. Similar to cholesterol and other fats in blood serum, a lower status of minerals such as zinc or magnesium may lead to increased Lp-PLA2 levels. Consequently, micronutrient deficiencies in MetS patients could potentially indicate higher risk factors for heart disease and stroke.

**Table 2:** Correlation analysis Lp-PLA2: Lipoprotein-Associated Phospholipase A2

Correlation	Pearson Correlation Coefficient (r)	p-value
Lp-PLA2 vs. Magnesium	-0.35	<0.001
Lp-PLA2 vs. Zinc	-0.42	<0.001

Comparison of groups

In comparison to the control group, individuals in the MetS group exhibited significantly higher serum Lp-PLA2 levels (239.8 ± 45.6 ng/mL vs. 178.3 ± 30.2 ng/mL, p < 0.001). Additionally, serum magnesium levels were significantly lower in the MetS group compared to controls (1.82 ± 0.14 mg/dL vs. 2.05 ± 0.18 mg/dL, p < 0.001), as were zinc levels (74.3 ± 9.6 µg/dL vs. 83.1 ± 10.4 µg/dL, p < 0.001), as given in Table 3. These differences underscore the potential impact of micronutrient deficiencies on cardiovascular health in individuals with MetS.

**Table 3:** Comparison of groups

Parameter	Metabolic Syndrome	Control	p-value
Lp-PLA2 (ng/mL)	239.8 ± 45.6	178.3 ± 30.2	<0.001
Magnesium (mg/dL)	1.82 ± 0.14	2.05 ± 0.18	<0.001
Zinc (µg/dL)	74.3 ± 9.6	83.1 ± 10.4	<0.001

Additional findings

Apart from these primary correlations, the study also looked at the relationship of Lp-PLA2 to several other cardiovascular markers. Lp-PLA2 was positively correlated to total cholesterol. In the MetS population, it r=0.29, p<0.01; and to LDL cholesterol., r=0.31; p<0.01, also in the Mets group. This suggests that more severe lipid abnormalities may accompany higher Lp-PLA2. In multi-regression analyses, further, Lp-PLA2 partially mediated the relationship between low serum magnesium and zinc levels and increased Atherogenic Index (β= 0.28, p<0.01), even when allowing for potential confounders such as age, sex, and BMI as given in Table 4. This mediating effect highlights the role of Lp-PLA2 in the complex interplay between micronutrient status and cardiovascular risk in MetS patients.

**Table 4:** Secondary findings

Parameter	Pearson Correlation Coefficient (r)	p-value
Lp-PLA2 vs. Total Cholesterol	0.29	0.01
Lp-PLA2 vs. LDL Cholesterol	0.31	0.01
Lp-PLA2 vs. Atherogenic Index	0.28	0.01

## Discussion

The results of this study show that serum magnesium and zinc levels are significantly negatively correlated with Lp-PLA2 in people with MetS. This suggests that a micronutrient shortage may exacerbate cardiovascular risk. In the MetS group, serum magnesium and zinc levels decreased. At the same time, Lp-PLA2 went up, a finding consistent with the large body of research reporting Lp-PLA2 as both an indicator for inflammation and a marker of atherosclerosis risk. Our results parallel other published observations showing higher Lp-PLA2 as a leading predictor of cardio events in groups such as Metabolic Syndrome patients [12]. However, our study sheds light on a new aspect, establishing connections between micronutrient shortage and Lp-PLA2 activity, offering new perspectives to nutritionally prepared end-stage disease patients with CVD who suddenly need fog-clearer medication.

Mechanistic insights

Consequently, while this cannot be conclusive evidence that their circulating levels of Lp-PLA2 control the metabolism of magnesium and zinc, it does look promising. Magnesium's important role in regulating vascular tone and endothelial function is well known; if magnesium is deficient, then the body's capacity to withstand oxidative stress and inflammation, two significant factors behind the development of atherosclerosis, will be reduced. Similarly, zinc is indispensable to acting as an antioxidant shield for cells and maintaining immune ability; once zinc is deficient, there is more likely to be increased oxidative damage and a very undesirable situation for repairing endothelium. Having more Lp-PLA2 in the blood could cause arteriosclerosis. By speeding up the hydrolysis of oxidized phospholipids into molecules that promote inflammation, the enzyme triggers endothelium inflammation and makes ruptured plaques susceptible to more clotting. Magnesium and zinc deficiencies could lead to an upregulation of Lp-PLA2 through LDL particles as they undergo extra oxidative stress, provoking further inflammatory responses [13]. This mechanistic pathway might account for MetS patients with low serum magnesium and zinc levels showing increased Lp-PLA2 concentrations.

Clinical implications

The findings have significant implications for clinical practice, especially in dealing with metabolic syndrome patients who are at higher risk of cardiovascular disease if diagnosed early enough. High Lp-PLA2 levels combined with low levels of magnesium and zinc form a helpful interface between probe and risk markers for cardiovascular events [14]. These differences underscore the potential impact of micronutrient deficiencies on cardiovascular health in individuals with MetS. Timely alterations of cardiovascular avoidable risk factors can be made if Lp-PLA2 levels are monitored in parallel with micronutrient supplementation to detect deficiencies at an early stage, along with general prevention strategies in treating patients with MetS. A relatively simple yet cost-effective means may be offered by nutritional supplementation of magnesium and zinc that is both able to offset some of the results generated from elevated Lp-PLA2--pro-inflammatory activity. Further clinical trials are needed to prove the usefulness of such an intervention.

Limitations

This study, however, has its limitations. A cross-sectional design means we cannot tell whether high Lp-PLA2 levels come before or follow micronutrient deficiencies. Longitudinal studies are required to confirm the direction of this relationship and observe the long-term effect on cardiovascular risk of correcting those inadequacies. Our study was conducted in a specific population. As such, the results may not be generalizable to other demographic groups or regions. Potential confounders such as dietary magnesium and zinc intake were not entirely controlled, which could affect the apparent correlations. Finally, Lp-PLA2 is well-established as an independent cardiovascular risk factor, and its precise role in precipitating complications in the development of MetS requires further research.

Recommendations

Future research should conduct longitudinal studies to establish a causal relationship between micronutrient levels and Lp-PLA2 activity in patients with MetS. Likewise, clinical trials that test whether magnesium or zinc supplementation can reduce Lp-PLA2 levels and, by extension, advance cardiac outcomes in patients who have this abnormal blood protein but are not symptomatic for heart disease yet are sorely needed. Research into the molecular mechanisms linking micronutrient shortages with elevated Lp-PLA2 activity could lead to more specific and compelling methods of lowering CVD risk in people with MetS. If it can be broadened to different populations, then studies like this may help to accumulate more evidence and draft nutritional policies specific to one region, which are recommended.

## Conclusions

This study points out that the Lp-PLA2 protein plays an important role in contrivance among patients with MetS who have micronutrient deficiency and cardiovascular risk. Our findings show that elevated levels of Lp-PLA2 are significantly associated with lower serum magnesium and zinc measures. This, in turn, increases the risk of heart disease events. These results reinforce the importance of micronutrient characteristics in evaluating cardiovascular risk among MetS patients. The reverse relationship between Lp-PLA2 and these microelements suggests that dietary therapies designed to correct magnesium and zinc deficiencies could be an innovation potentially useful for cutting cardiovascular risk as they affect inflammatory pathways.

## References

[1] Silveira Rossi JL, Barbalho SM, Reverete de Araujo R, Bechara MD, Sloan KP, Sloan LA (2022). Metabolic syndrome and cardiovascular diseases: Going beyond traditional risk factors. Diabetes Metab Res Rev.

[2] Aguilar-Salinas CA, Viveros-Ruiz T (2019). Recent advances in managing/understanding the metabolic syndrome. F1000Res.

[3] Mohammadifard N, Humphries KH, Gotay C (2019). Trace minerals intake: Risks and benefits for cardiovascular health. Crit Rev Food Sci Nutr.

[4] Mancuso E, Perticone M, Spiga R (2020). Association between serum Mg(2+) concentrations and cardiovascular organ damage in a cohort of adult subjects. Nutrients.

[5] Lee SR (2018). Critical role of zinc as either an antioxidant or a prooxidant in cellular systems. Oxid Med Cell Longev.

[6] Dragan S, Buleu F, Christodorescu R (2019). Benefits of multiple micronutrient supplementation in heart failure: A comprehensive review. Crit Rev Food Sci Nutr.

[7] Wang C, Fang X, Hua Y (2018). Lipoprotein-associated phospholipase A2 and risk of carotid atherosclerosis and cardiovascular events in community-based older adults in China. Angiology.

[8] Virmani R, Kolodgie FD, Burke AP (2005). Atherosclerotic plaque progression and vulnerability to rupture: angiogenesis as a source of intraplaque hemorrhage. Arterioscler Thromb Vasc Biol.

[9] Hu J, Lei H, Liu L, Xu D (2022). Lipoprotein (a), a lethal player in calcific aortic valve disease. Front Cell Dev Biol.

[10] Baziar N, Nasli-Esfahani E, Djafarian K (2020). The beneficial effects of alpha lipoic acid supplementation on Lp-PLA2 mass and its distribution between HDL and apoB-containing lipoproteins in type 2 diabetic patients: a randomized, double-blind, placebo-controlled trial. Oxid Med Cell Longev.

[11] Kumari S, Kataria DK, Kumari S (2024). Metabolic syndrome frequency in type 2 diabetics using International Diabetes Federation (IDF) criteria analysis. Cureus.

[12] Mallela SK, Puranam K, Neelam S (2024). Predictive value of Lp-PLA2 for coronary artery disease in type 2 diabetes mellitus patients: an observational study. Inter Jr Diab Dev Con.

[13] Liu M, Dudley SC Jr (2020). Magnesium, oxidative stress, inflammation, and cardiovascular disease. Antioxidants (Basel).

[14] Zhu S, Wei X, Yang X (2019). Plasma lipoprotein-associated phospholipase A2 and superoxide dismutase are independent predicators of cognitive impairment in cerebral small vessel disease patients: diagnosis and assessment. Aging Dis.

